# Two Types of Morphologically Distinct Fibers Comprising *Gallionella ferruginea* Twisted Stalks

**DOI:** 10.1264/jsme2.ME11340

**Published:** 2012-03-28

**Authors:** Tomoko Suzuki, Hideki Hashimoto, Hiromichi Ishihara, Nobuyuki Matsumoto, Hitoshi Kunoh, Jun Takada

**Affiliations:** 1 Department of Material Chemistry, Graduate School of Natural Science and Technology, Okayama University, 3–1–1 Tsushima-naka, Kita-ku, Okayama 700–8530, Japan

**Keywords:** *Gallionella ferruginea*, iron-oxidizing bacteria, organic/inorganic hybrid, two stalk fibers

## Abstract

Two morphologically distinct extracellular stalk fibers produced by *Gallionella ferruginea* were compared by electron microscopy and elemental analysis. The thick- and fine-fiber stalks were different in structure on a micrometer scale and in the site on the mother cell to which they were attached, but on a nanometer scale they were similar in ultrastructure and in the elemental composition of their basic fiber matrix.

Bacteria belonging to the genus *Gallionella* produce uniquely twisted extracellular stalks and are ubiquitous inhabitants of ocherous deposits that form in freshwater bodies (2). This genus has had an anfractuous history of nomenclature, as described in detail by Silva (7), but the current genus name has gained acceptance by most researchers (2). Although several names for this species have been proposed since the beginning of the 19^th^ century, the term *Gallionella ferruginea* has been commonly used for the most ubiquitous species found worldwide. Despite some past and present debates, it seems conceivable that the genus may consist of only a single species (*G. ferruginea*) and all the other described species are only ecological variants of it (5); however, much of the detailed taxonomical position of this genus is still obscure because of the lack of isolated strains. Søgaard *et al.*(8) used microscopy to compare iron (Fe) sludge from filter systems belonging to three different Danish freshwater plants and found that the stalks of *G. ferruginea* collected from one plant were composed of small, twisted parallel threads, whereas those from the other two plants were larger and typically twisted, suggesting that the two types of stalks might belong to different species. Several papers (2, 4, 6, 8, 10) have reported the ultrastructure of *G. ferruginea* cells and their associated stalks but the structural and elemental associations of the cells and stalks are still not well understood.

In this study, we found two *Gallionella*-type twisted stalks composed of morphologically distinct fibers that coexisted in the same freshwater purification plant. These stalk fibers were observed at nearly the same proportion in the plant. In this paper, we compared the detailed structural and elemental similarities and differences between these two types of fibers using electron microscopy and elemental analyses.

Ocherous flocs associated with microbial mats attached to the walls of a groundwater-receiving tank were collected from a freshwater purification pilot plant at Okayama University farm. Typically twisted stalks produced by *G. ferruginea* were confirmed by light microscopy as the predominant deposits in the flocs (9).

For microscopic examinations, deposit samples were prepared according to a protocol described previously (9). Briefly, for transmission electron microscopy (TEM), the deposit was fixed with a mixture of 2.5% glutaraldehyde, 1% OsO_4_, and 4.5% sucrose in 100 mM cacodylate buffer (pH 7.0) for 2 h on ice, and then embedded in 2% agar. Small pieces of the agar block were then stained with 0.5% uranyl acetate for 15 min, and dehydrated in a graded ethanol series after repeated washing and embedded in a resin mixture (Quetol 651; Nisshin EM, Tokyo, Japan). Ultrathin sections were observed by TEM (H-7500; Hitachi, Tokyo, Japan) at 80 kV or scanning transmission electron microscopy (STEM) (JEM-2100F; JEOL, Tokyo, Japan) with a spherical aberration *C*_s_ corrector (CEOS, Heidelberg, Germany) at 200 kV. To observe the elemental localization on the ultrathin sections, unstained ultrathin sections mounted on copper (Cu) grids covered with a formvar film and a carbon (C) coat were subjected to high-angle annular dark-field STEM (HAADF-STEM) and energy dispersive X-ray spectroscopy (EDX) elemental mapping, using a TEM equipped with an EDX detector (JED-2300T, JEOL). For STEM/secondary electron imaging (STEM/SEI) and STEM electron energy-loss spectroscopy (EELS) analyses, a suspension of the washed specimens was mounted onto Cu grids pre-coated with an amorphous lacy C film (Nisshin EM). For scanning electron microscopy (SEM) (S-800 or S-4300; Hitachi), the suspension was dropped onto an aluminum (Al) stub, vacuum dried, and platinum (Pt)-coated.

Generally, the fibers arise from the cell surface and stretch over 2 μm from the cell, followed by entire twining to result in a typical twisted stalk; therefore, the stalk is roughly partitioned in the stretching and twisting regions (Fig. 1A). Careful SEM and TEM observations led us to identify two types of uniquely twisted *Gallionella* stalks in the ocherous flocs. One type (Fig. 1B) was a stalk with fine fibers attached to a narrow portion of the concave side of a kidney-shaped bacterial cell; it comprised an assembly of electron-dense, extremely fine fibers (hereafter referred to as F-fiber). The other type (Fig. 1C) had much thicker stalk fibers that arose entirely from one side of the cell (hereafter referred to as T-fiber). Regarding the attachment zone of the fibers to the apical cell, the F-fiber stalk resembled the *Gallionella* stalk described by Ghiorse (2), while the T-fiber stalk was quite similar to that of *Mariprofundus ferrooxydans* by Chan *et al.*(1) and *G. ferruginea* by Suzuki *et al.*(9).

Comparisons of the surface morphology of these two fiber types by SEM and STEM/SEI were as follows: i) Stalk width was almost identical in both types (1.25±0.16 and 0.95±0.11 μm on average in 50 F- and T-fiber stalks, respectively) (Fig. 2A and D). ii) The F-fiber stalks were entirely twisted over 2 μm from the cell (Fig. 2A) and were composed of a networked assembly of stretching, curved, or branching fine fibers (width of 50 fibers in SEM images ranged 5–20 nm) (Fig. 2B and C). The T-fibers were twisted and arrayed in parallel (Fig. 2D) but their constituent fibers were much thicker (20–100 nm thick) (Fig. 2E and F). iii) Morphological differences between stalk fibers were observed on the micrometer scale whereas the nanometer scale structures of both fibers were very similar, as the surface of both fibers was rough and granular (Fig. 2C and F).

EDX applied to thin sections of the F-fiber stalks detected major peaks for oxygen (O) and Fe, and minor peaks for phosphorus (P) and silicon (Si) (Fig. S1A). These findings are similar to those for T-fiber stalks described in a previous paper (9). The intensity of the detected P and Si signals was insufficient for distribution mapping, unlike in the previous paper. Fig. S1B shows the merged HAADF-STEM images and EDX map of Fe distribution (red signals) in the F-fiber stalks, and appears to indicate that Fe is distributed in the fibers but not in the apical cells. Similarly, elemental mapping applied to TEM image of T-fibers in a previous paper (9) revealed that Fe distribution was restricted to the fibers but not the apical cells. A single fiber from the F-fiber stalk was selected from the framed HAADF-STEM image (Fig. 3A and B) as a target for EELS analysis (Fig. 3C, D, E and F). In the high power HAADF-STEM image (Fig. 3B), the darker spots of varying sizes in the rather smooth, bright region most likely reflect structural unevenness in the fiber matrix, which is consistent with the image from T-fiber stalks (9). On the EELS maps, fine, bright signal spots represent sites of energy loss of the incident electron beam by the elements present. Because the energy loss by the respective elements occurs at specific electron voltages, the precise nanometer-level localization of the specific element is defined by comparing the site density with the background darkness produced by the lack of the target element. Thus, the observation of some darker regions (Fig. 3C, D, E and F) indicates a lower density but not the absence of the target element (9). The localization patterns of C, O, and Fe in the F-fiber stalk matrix were fundamentally the same as those in the T-fiber stalk (9). C distribution (Fig. 3C) was characterized by numerous bright signals, reflecting a high concentration. Intense C signals were not detected in the marginal fiber region, as was evident by comparing the image in Fig. 3C with that in Fig. 3B, which was obtained from the same framed area in Fig. 3A at the same magnification. This localization suggests that C could exist in the core but to a lesser degree, or not at all, at the margin of the fiber. Similarly, in the T-fiber stalks (9), both O (Fig. 3D) and Fe (Fig. 3E) co-localized more evenly with fewer low-density spots than C in the entire fiber matrix, suggesting that Fe could exist as iron oxide and/or oxyhydroxide in the fiber. The merged image of the C/Fe maps (Fig. 3F) illustrates the localization of C (green) in the fiber core and Fe (red) throughout the fiber. This image led us to conclude that the fiber could consist of structural units that might form an intermingled and folded C fiber network (Fig. 3A) of bacterial saccharic materials probably excreted from bacterial cells; this could interact with aqueous Fe. It seems likely that the residual O detected throughout the fiber might also interact with other elements, including C, Si, and P.

In conclusion, the two types of stalk fibers were morphologically distinct in fiber width at the micrometer level. In addition, the F-fibers were attached to a narrow region on the concave side of an apical cell whereas the T-fibers were attached to almost the entire length of one side of an apical cell; however, the basic matrix of both types was similar in terms of nanometer-scale structures and elemental localization.

Although at present it is not known whether the mother cells might belong to a species different from *G. ferruginea*, as Søgaard *et al.*(8) suggested, or whether they represent different ecological forms of this species, as was suggested by Hanert (5), we tentatively regarded them as *G. ferruginea* in this paper, because to date the characteristic twisted stalk is the only identification mark of *Gallionella*(3). Here, we would like to emphasize that two morphologically distinct extracellular stalk fibers are produced by so-called *G. ferruginea*. This paper leaves the above queries unsolved. To solve these queries, separate strains which produce these two types of stalk fiber must be isolated and detailed phylogenic and genetic analyses, such as a combination of single cell genomics and very stringent FISH, are apparently required. For this purpose we are attempting to isolate *Gallionella*, which is generally known as the most difficult bacterium to isolate from nature.

## Supplemental materials



## Figures and Tables

**Fig. 1:**
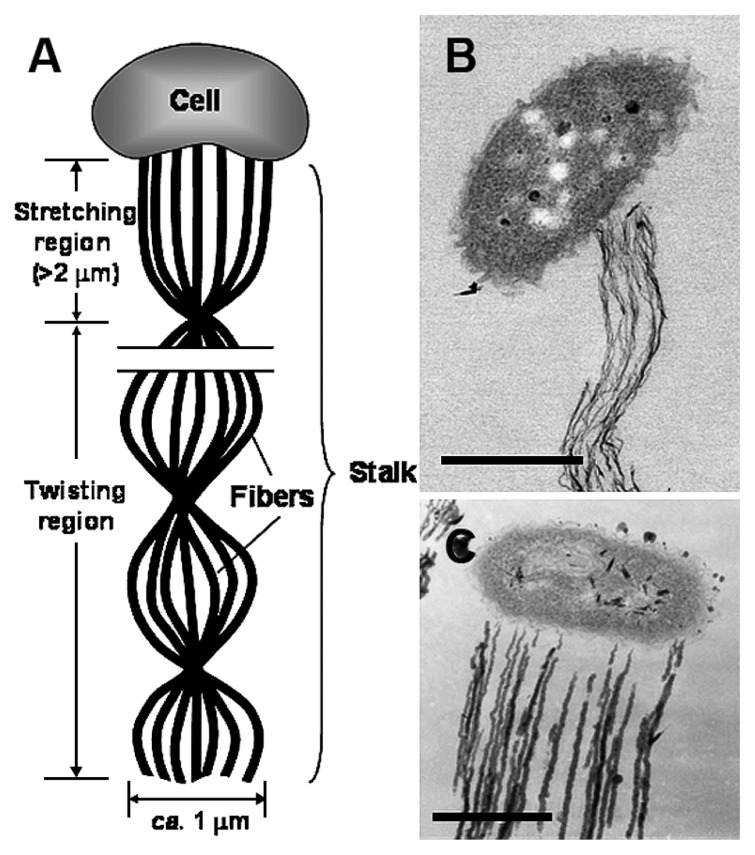
Schematic model of twisted stalk from *Gallionella* cell (A). STEM (B) and TEM (C) images of the two types of *G. ferruginea* stalk. (B) Fine fibers comprising a networked assembly at the stretching region of the stalk (F-fiber stalk). The stalk arises from a narrow region on the concave side of an apical bacterial cell. (C) Thick fibers comprising a parallel array in the stretching region of the stalk (T-fiber stalk). Note that the stalk arises from a broad region that covers nearly the entire length of one side of the apical cell. Bars, 500 nm.

**Fig. 2:**
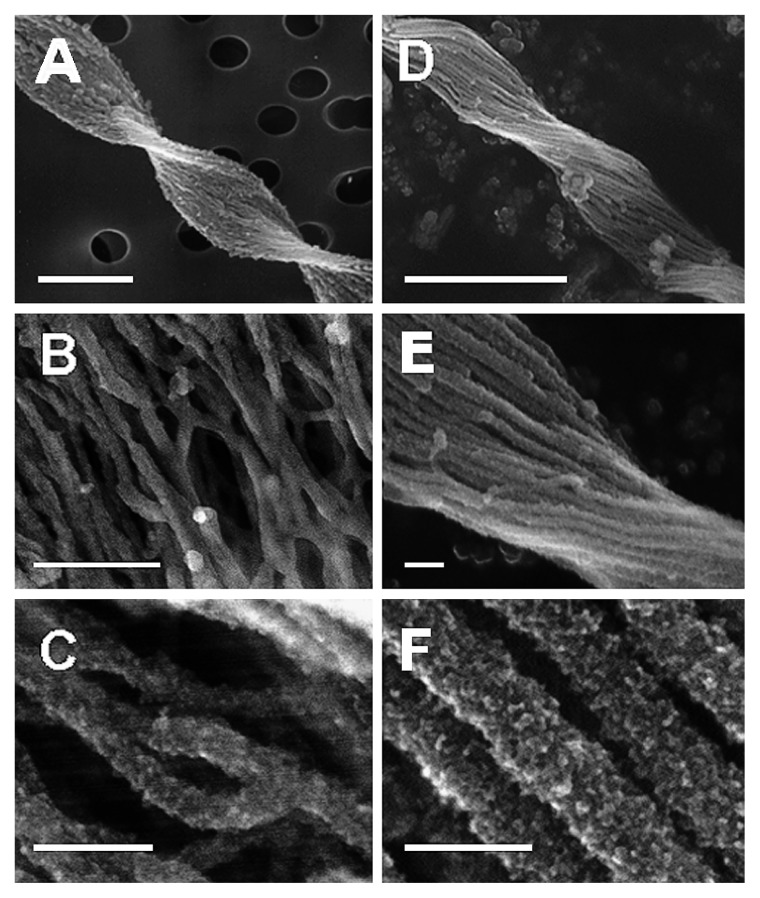
SEM and STEM/SEI images of F- and T-fiber stalks. (A) A typically twisted F-fiber stalk. Bar, 2 μm. (B) A network of straight, curved, and branching fine fibers of the F-fiber stalk. Bar, 100 nm. (C) High-power image of a network of fine fibers in the F-fiber stalk. Bar, 50 nm. (D) A typically twisted T-fiber stalk. Bar, 2 μm. (E, F) Parallel array of thick fibers in the T-fiber stalk. Bars (E), 100 nm and (F), 50 nm.

**Fig. 3:**
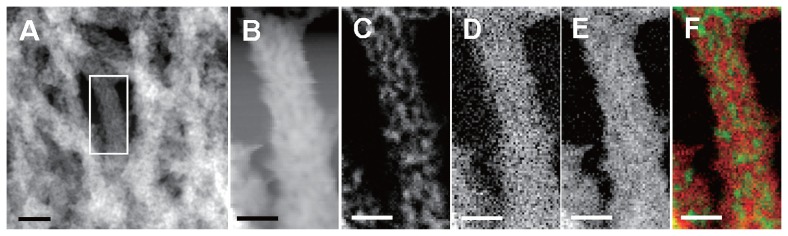
HAADF-STEM images of the F-fiber stalk and STEM-EELS maps of Fe, O, and C in a single fiber. (A) HAADF-STEM image of a stalk composed of networked fibers. Bar, 20 nm. (B) Enlarged HAADF-STEM image of a single fiber from the boxed area shown in panel A, on which EELS mapping was performed. (C) EELS spectrum image of the C-K edge. Note that intense C signals are concentrated in the core fiber regions and not at the marginal edge. (D, E) EELS spectrum image of the O-K and Fe-L_2,3_ edges, respectively. Both elements were distributed relatively evenly, with some darker spots caused by lower density and not by absence. (F) Merged images from panels C and E. Note the co-localization of C (green) in the fiber core and Fe (red) throughout the fiber, even in the marginal region. Bars (B to F), 10 nm.
