# Supplementary material for: Two Types of Morphologically Distinct Fibers Comprising Gallionella ferruginea Twisted Stalks
Source: Microbes Environ. 2012 Mar 28;27(3):338–41. doi: 10.1264/jsme2.ME11340 (PMC4036057; doi:10.1264/jsme2.ME11340)
Supplement: Supplementary file 1 [file 27_338_s1.pdf]

Supplemental materials

Fig. S1. STEM-EDX analysis of sectioned F-fiber stalks. (A) An EDX spectrum showing major peaks of C, O, and Fe and minor peaks of P and Si. Peaks of uranium (U) and Cu are attributable to the stain and grid used, respectively. (B) Merged HAADF-STEM image of an enlarged area of Figure 1A and Fe distribution map. Note that Fe (red color) is localized in the fibers but not in the apical bacterial cell. Bar, 100 nm.

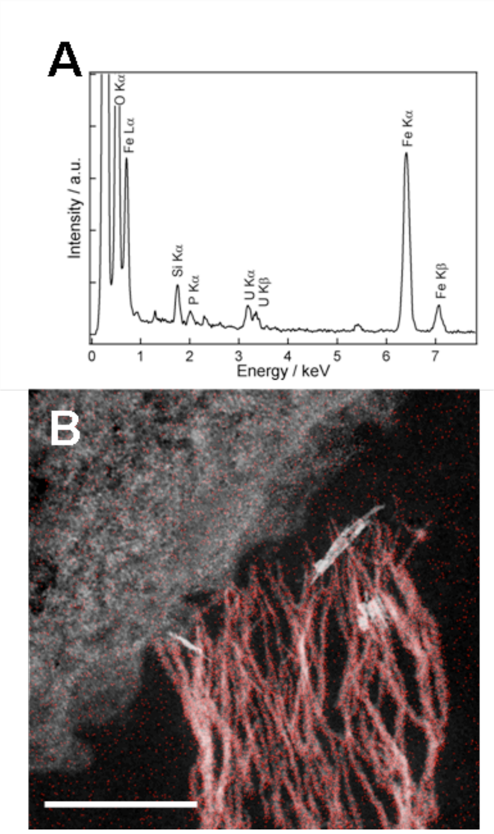
A
O Kα
Fe Lα
Si Kα
P Kα
U Kα
U Kβ
Fe Kα
Fe Kβ
Intensity / a.u.
0
1
2
3
4
5
6
7
Energy / keV
B

Fig. S1
